# Association between triglyceride-glucose index and treatment response in primary membranous nephropathy: a retrospective cohort study

**DOI:** 10.1080/0886022X.2026.2751267

**Published:** 2026-11-04

**Authors:** Wei Chen, Tan Hou, Weimin Yu

**Affiliations:** Third Hospital of Shanxi Medical University, Shanxi Bethune Hospital, Shanxi Academy of Medical Sciences, Tongji Shanxi Hospital, Taiyuan, Shanxi, China

**Keywords:** Membranous nephropathy, triglyceride-glucose index, proteinuria remission, anti-PLA2R antibody, prognosis

## Abstract

**Background/Objectives:**

The triglyceride-glucose (TyG) index, a marker of insulin resistance, has been linked to chronic kidney disease and proteinuria severity, but its prognostic value for treatment response in primary membranous nephropathy (PMN) is unclear. We evaluated whether baseline TyG predicts 12-month proteinuria remission in PMN.

**Methods:**

We included 363 patients with PMN. The primary outcome was 12-month remission per 2021 KDIGO criteria. Missing covariates were handled by multiple imputation (20 datasets). Multivariable logistic regression adjusted for anti-PLA2R antibody (continuous, per 100 RU/mL), albumin, 24-hour urine protein, age, sex, comorbidities, and treatment group. Sensitivity analyses included TyG tertiles, restriction to standard immunotherapy, complete remission, binary anti-PLA2R status, eGFR adjustment, log-transformed and restricted cubic spline modeling of anti-PLA2R antibody, complete-case analysis, inverse probability of treatment weighting, post-hoc power analysis, and adjustment for treatment switching.

**Results:**

Among 363 patients, 267 (73.6%) achieved remission. Baseline TyG was not independently associated with remission (OR = 0.839, 95% CI: 0.544–1.293, *p* = 0.425). Higher anti-PLA2R antibody levels were associated with lower odds of remission when modeled continuously (OR per 100 RU/mL = 0.836, 95% CI: 0.704–0.992, *p* = 0.041), but not when dichotomized (OR = 0.666, 95% CI: 0.213–2.089, *p* = 0.484). Addition of TyG minimally improved model discrimination (AUC 0.714 to 0.716). Results were consistent across sensitivity analyses.

**Conclusions:**

Baseline TyG was not independently associated with 12-month remission in PMN and provided minimal incremental predictive value. However, a moderate association cannot be excluded given the confidence interval. Anti-PLA2R antibody level showed an inverse association when modeled continuously.

## 1. Introduction

Primary membranous nephropathy (PMN) is one of the most common causes of nephrotic syndrome in adults [1], accounting for 20% to 37% of cases in most series and up to 40% among adults over 60 years of age [2]. Its clinical course is heterogeneous, ranging from spontaneous remission to persistent nephrotic syndrome and progressive kidney dysfunction [3]. Early identification of patients at high risk for poor outcomes is therefore important for individualized treatment and monitoring.

The discovery of anti-phospholipase A2 receptor (PLA2R) antibodies has substantially advanced the diagnosis and prognostic assessment of PMN [4,5]. The 2021 Kidney Disease: Improving Global Outcomes (KDIGO) guideline recognizes serum anti-PLA2R antibodies as an important diagnostic and monitoring biomarker in patients with PMN [6]. Nevertheless, treatment response remains influenced by multiple factors, including baseline proteinuria, serum albumin, kidney function, antibody burden, and treatment strategy [7–9].

Increasing evidence suggests that metabolic abnormalities may be relevant to kidney disease [10]. The triglyceride-glucose (TyG) index is a readily available surrogate marker of insulin resistance calculated from fasting triglyceride and glucose concentrations [11,12]. Previous studies have reported associations between TyG and chronic kidney disease, proteinuria, and adverse renal outcomes [13,14]. A recent study in patients with PMN also reported associations between higher TyG and more severe proteinuria and impaired renal function [15].

However, most available evidence has focused on cross-sectional associations between TyG and disease severity rather than longitudinal treatment response. Whether baseline TyG provides prognostic information for proteinuria remission after treatment in PMN remains uncertain. In particular, it is unclear whether TyG provides additional predictive information beyond established clinical and immunological markers such as anti-PLA2R antibody, serum albumin, proteinuria, and treatment strategy.

Insulin resistance and metabolic dysregulation may influence treatment response through multiple mechanisms, including modulation of immune cell function and podocyte injury [16,17]. Therefore, although TyG has been primarily studied as a marker of disease severity, it is biologically plausible that it may also affect treatment response. However, this hypothesis has not been directly tested in PMN: most available evidence has focused on cross-sectional associations between TyG and disease severity rather than longitudinal treatment response. Whether baseline TyG provides prognostic information for proteinuria remission after treatment in PMN, particularly beyond established clinical and immunological markers such as anti-PLA2R antibody, serum albumin, proteinuria, and treatment strategy, remains uncertain.

Therefore, we conducted a retrospective cohort study to evaluate the association between baseline TyG and 12-month proteinuria remission in patients with PMN and to determine whether incorporation of TyG improves predictive performance beyond conventional clinical and treatment-related variables.

## 2. Methods

### 2.1. Study design and participants

This single-center retrospective cohort study was conducted at the Department of Nephrology, Shanxi Bethune Hospital. The study protocol was approved by the institutional ethics committee (approval number: NO.LYLL-2026-006/PJ160). Written informed consent was waived because of the retrospective design and use of anonymized clinical data.

We screened electronic medical records of adult patients diagnosed with PMN between January 1, 2015, and June 30, 2025. Eligible patients met at least one of the following diagnostic criteria: positive serum anti-PLA2R antibody in the appropriate clinical setting or biopsy-proven PMN in antibody-negative patients. Additional eligibility criteria included age ≥18 years and availability of sufficient baseline and follow-up information for outcome assessment. Prespecified exclusion criteria included immunosuppressive therapy for more than 2 weeks before enrollment, secondary membranous nephropathy, baseline eGFR <15 mL/min/1.73 m^2^ or dialysis, and active infection, malignancy, or active autoimmune disease. During retrospective screening, only 1 of 364 patients was excluded (age <18 years); all remaining patients met the inclusion criteria and did not fulfill any prespecified exclusion criteria. The final analysis included 363 patients. The data cutoff date was June 30, 2025.

### 2.2. Data collection

Baseline was defined as the most recent measurement within 2 weeks after diagnosis and before initiation of immunosuppressive therapy. Baseline demographic characteristics included age, sex, smoking history, alcohol consumption, diabetes mellitus, hypertension, and cardiovascular disease. Laboratory variables included complete blood count parameters, serum albumin, creatinine, fasting blood glucose, triglycerides, total cholesterol, high-density lipoprotein cholesterol, low-density lipoprotein cholesterol, alanine aminotransferase, aspartate aminotransferase, electrolytes, 24-h urine protein, complement C3 and C4, immunoglobulin G, and serum anti-PLA2R antibody levels.

Treatment regimens were categorized according to the initial immunosuppressive strategy into five groups:

1. **CTX**: cyclophosphamide combined with glucocorticoids;
2. **CNI**: tacrolimus or cyclosporine combined with glucocorticoids;
3. **RTX**: rituximab with or without glucocorticoids;
4. **Other**: initial combination regimens consisting of glucocorticoids plus a calcineurin inhibitor and rituximab, or glucocorticoids plus cyclophosphamide and rituximab, which did not fit into the CTX, CNI, or RTX monotherapy-based categories;
5. **Conservative**: supportive care with renin-angiotensin system inhibitors and/or glucocorticoids alone, without other immunosuppressants.

Patients who switched regimens during follow-up were assigned to their initial treatment category. The proportion of patients who switched regimens is reported in Table 1. In sensitivity analyses, we additionally adjusted for treatment switching as a binary covariate and performed a subgroup analysis restricted to patients who did not switch regimens. Follow-up measurements included 24-h urine protein, serum albumin, and kidney function at 12 months.

**Table 1.** Baseline characteristics of the study population.

Variable	Overall (*N* = 363)	Non-remission (*N* = 96)	Remission (*N* = 267)	*p* value
Age, years	51.37 (12.67)	51.59 (12.25)	51.28 (12.84)	0.838
eGFR, mL/min/1.73 m²	96.38 (18.47)	94.91 (19.13)	96.91 (18.23)	0.365
Serum albumin, g/L	25.40 (6.15)	24.03 (6.86)	25.89 (5.80)	**0.011**
24-hour urine protein, g/d	6.30 (4.01)	7.20 (5.27)	5.97 (3.39)	**0.010**
Anti-PLA2R, median (IQR), RU/mL	47.75 (23.05–113.98)	58.87 (24.91–188.94)	45.40 (21.40–95.00)	**<0.001**
TyG index	9.20 (0.62)	9.27 (0.63)	9.18 (0.62)	0.203
NLR	3.17 (5.04)	2.46 (1.84)	3.42 (5.76)	0.110
Male, n (%)	235 (64.7)	66 (68.8)	169 (63.3)	0.404
Diabetes, n (%)	46 (12.7)	14 (14.6)	32 (12.0)	0.633
Hypertension, n (%)	155 (42.7)	43 (44.8)	112 (41.9)	0.717
Cardiovascular disease, n (%)	17 (4.7)	7 (7.3)	10 (3.7)	0.259
Treatment, n (%)				**0.002**
CTX	29 (8.0)	5 (5.2)	24 (9.0)	
CNI	165 (45.5)	52 (54.2)	113 (42.3)	
RTX	102 (28.1)	14 (14.6)	88 (33.0)	
Other	27 (7.4)	8 (8.3)	19 (7.1)	
Conservative	40 (11.0)	17 (17.7)	23 (8.6)	
Treatment switch, n (%)	78 (21.5)	17 (17.7)	61 (22.8)	0.365

Data are presented as mean ± SD or median (IQR) for continuous variables and n (%) for categorical variables. Anti-PLA2R antibody levels are presented as median (IQR) due to right-skewed distribution.

### 2.3. Anti-PLA2R antibody handling

Anti-PLA2R antibody was measured using a commercial enzyme-linked immunosorbent assay (ELISA) kit. The same assay platform and kit were used throughout the study period. Anti-PLA2R antibody was prespecified as a continuous exposure in the primary analysis and was expressed per 100 RU/mL (PLA2R_per100). Values reported by the assay as <2 RU/mL were coded as 1 RU/mL for the continuous analysis according to the prespecified analytical convention, representing the lowest measurable value. This approach retained all observations in the continuous model without treating below-detection measurements as missing. In a separate sensitivity analysis, anti-PLA2R antibody status was dichotomized as negative (<2 RU/mL) versus positive (≥2 RU/mL).

### 2.4. Outcomes

The primary outcome was 12-month proteinuria remission, defined according to the 2021 KDIGO guideline [6]. Complete remission was defined as 24-h urine protein <0.3 g/d, serum albumin ≥35 g/L, and stable kidney function. Partial remission was defined as *a* ≥ 50% reduction in 24-h urine protein from baseline to a final value <3.5 g/d, serum albumin ≥30 g/L, and stable kidney function. Stable kidney function was defined as eGFR decline <15% from baseline. The 12-month assessment window was 12 ± 1 months. Patients who met either complete or partial remission criteria were classified as having overall remission, whereas patients who met neither criterion were classified as having non-remission. Complete remission was evaluated as a secondary outcome in sensitivity analysis.

### 2.5. Definitions of key exposures

TyG was calculated according to the standard formula: TyG = ln[TG (mg/dL) × FBG (mg/dL)/2]. Because triglycerides and fasting blood glucose were recorded in mmol/L, triglycerides were converted to mg/dL by multiplying by 88.57 and glucose by 18.02 before calculation. If either triglyceride or fasting glucose was ≤0, TyG was considered missing. NLR was calculated as the neutrophil count divided by the lymphocyte count.

Using the standard formula and the converted mg/dL concentrations, the TyG index in the present cohort was approximately 9.20 ± 0.62 overall, 9.18 ± 0.62 among patients who achieved remission, and 9.27 ± 0.63 among those who did not achieve remission.eGFR was calculated using the 2021 CKD-EPI creatinine equation without a race coefficient [18]: eGFR = 142 × min(SCr/κ,1)^α × max(SCr/κ,1)^−1.200 × 0.9938^Age × 1.012 (if female), where κ = 0.7 for women and 0.9 for men, α= −0.241 for women and −0.302 for men, and serum creatinine was converted from μmol/L to mg/dL by dividing by 88.4.

### 2.6. Missing data

Missing baseline covariates were handled using multiple imputation by chained equations with 20 imputed datasets and 20 iterations per dataset [19]. Predictive mean matching was used for continuous variables, while logistic or multinomial regression was used for binary or categorical variables, as appropriate. The imputation model incorporated the baseline clinical and laboratory variables included in the analytical framework together with relevant auxiliary variables. Follow-up measurements used to define the outcome were not imputed when corresponding outcome information was unavailable. Remission outcomes were derived deterministically from the available 12-month proteinuria, serum albumin, and kidney function measurements. The proportion of missing data for each variable is reported in Table S1. MICE convergence plots are provided in Figure S1.

### 2.7. Statistical analysis

All analyses were performed using R version 4.4.2 (R Foundation for Statistical Computing). Continuous variables were summarized as mean ± standard deviation or median (interquartile range), according to their distribution, and categorical variables were summarized as counts and percentages. Baseline characteristics were compared between patients with and without 12-month remission using the Student’s t-test, Wilcoxon rank-sum test, or chi-square test, as appropriate. Two-sided *p* < 0.05 was considered statistically significant.

Four nested multivariable logistic regression models were constructed. Model 1 included baseline PLA2R_per100, serum albumin, 24-h urine protein, age, sex, diabetes, hypertension, and cardiovascular disease. Model 2 additionally included treatment group. Model 3 further included TyG and was designated as the primary TyG model. Model 4 additionally included NLR as an exploratory extension. CTX was used as the reference category for treatment group. Anti-PLA2R antibody was modeled continuously as PLA2R_per100, corresponding to a 100-RU/mL increase.

Prespecified sensitivity analyses included: TyG tertiles, restriction to standard immunotherapy recipients, complete remission as outcome, and binary anti-PLA2R status. Additional sensitivity analyses were performed: (1) adding baseline eGFR to Model 3; (2) modeling anti-PLA2R antibody as log-transformed and restricted cubic splines; (3) complete-case analysis; (4) inverse probability of treatment weighting (IPTW) comparing standard immunotherapy versus conservative treatment; (5) post-hoc power analysis; and (6) including treatment switching as a binary covariate in Model 3 and performing a subgroup analysis restricted to patients who did not switch regimens. The DeLong test was used to compare AUCs between Model 2 and Model 3.

Regression estimates from the 20 imputed datasets were combined using Rubin’s rules [20]. Odds ratios (ORs), 95% confidence intervals (CIs), and two-sided *p* values were reported.

### 2.8. Model performance

For descriptive purposes, model discrimination was assessed using the area under the receiver operating characteristic curve (AUC), and model fit was summarized using Akaike’s information criterion (AIC). AUC and AIC were calculated separately within each imputed dataset and summarized as mean values across the 20 imputations. Because formal Rubin-style inference is not straightforward for these measures, comparisons were considered exploratory rather than confirmatory. Variance inflation factors (VIFs) were examined to evaluate potential multicollinearity. ROC curves for all four models are provided in Figure S2.

## 3. Results

### 3.1. Study population

A total of 364 patients were initially screened. One patient was excluded because of age <18 years; no patients were excluded for any other prespecified criterion. The final analysis included 363 patients. All included patients had 12-month outcome data. A study flow diagram is presented in Figure 1. The mean age was 51.4 ± 12.7 years, and 235 (64.7%) patients were male. Anti-PLA2R antibody was positive (≥2 RU/mL) in 331 patients (91.2%) and negative (<2 RU/mL) in 32 patients (8.8%). Treatment included CNI-based therapy in 165 patients (45.5%), RTX in 102 (28.1%), CTX in 29 (8.0%), other combination regimens in 27 (7.4%), and conservative treatment in 40 (11.0%). Overall, 267 patients (73.6%) achieved 12-month proteinuria remission, including 107 (29.5%) who achieved complete remission.

**Figure 1.**
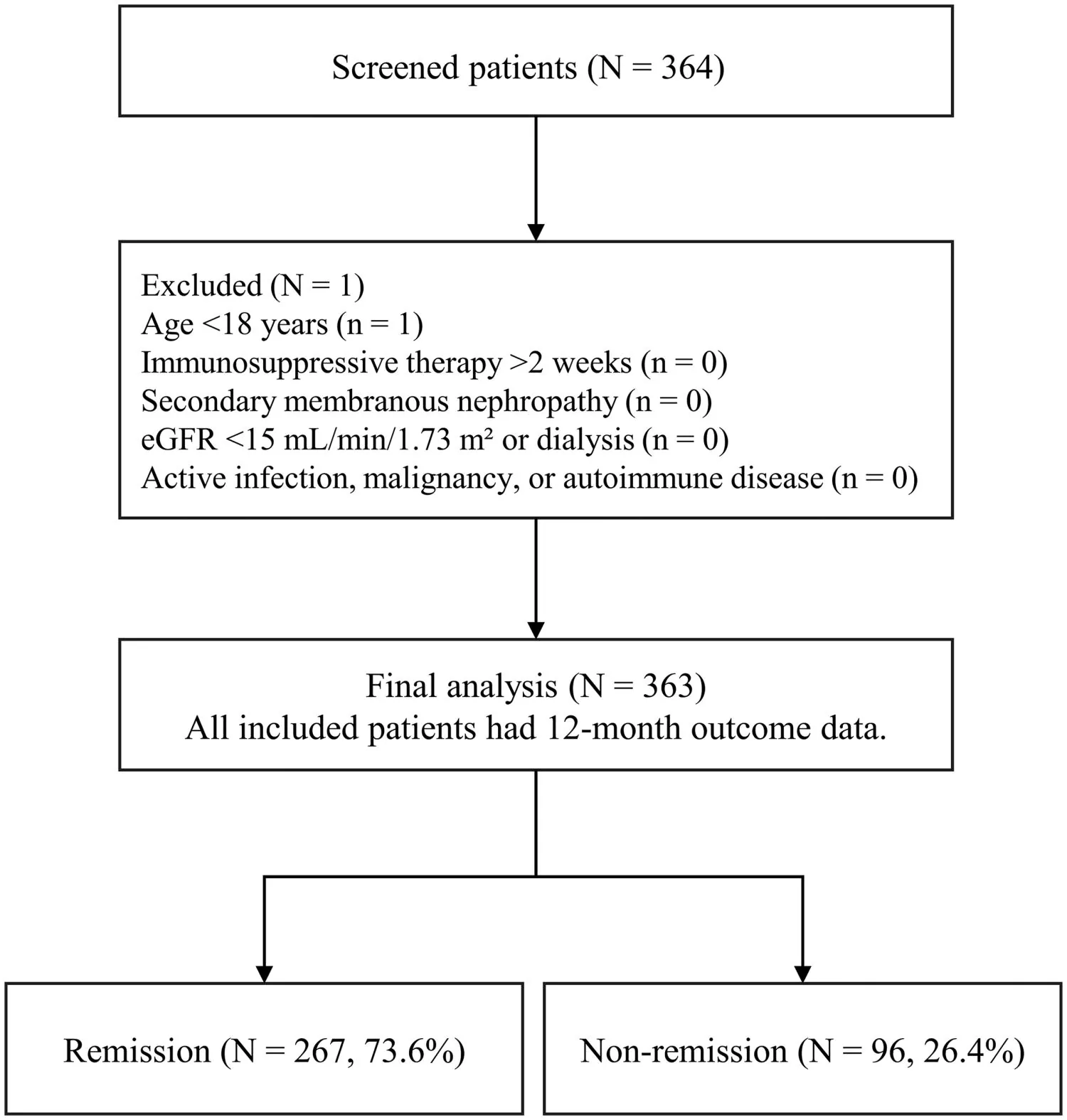
Study flow diagram.

Patients who achieved remission had higher baseline serum albumin (25.89 vs 24.03 g/L, *p* = 0.011) and lower baseline 24-h urine protein (5.97 vs 7.20 g/d, *p* = 0.010) and anti-PLA2R antibody levels (median [IQR]: 45.40 [21.40–95.00] vs 58.87 [24.91–188.94] RU/mL, *p* < 0.001) than those who did not achieve remission. The TyG index was numerically lower among patients who achieved remission (9.18 vs 9.27), but the between-group difference was not statistically significant (*p* = 0.203). A total of 78 patients (21.5%) switched treatment regimens during follow-up. The switch proportion was 17/96 (17.7%) in the non-remission group and 61/267 (22.8%) in the remission group (*p* = 0.365). Switch proportions by initial treatment group were: CTX 9/29 (31.0%), CNI 32/165 (19.4%), RTX 25/102 (24.5%), Other 4/27 (14.8%), and Conservative 8/40 (20.0%). The detailed baseline characteristics are presented in Table 1.

### 3.2. Multivariable logistic regression

In the primary TyG model (Model 3), baseline TyG was not independently associated with 12-month remission (OR = 0.839, 95% CI: 0.544–1.293, *p* = 0.425). Although the point estimate was below 1.0, the confidence interval included the null value. Given the wide confidence interval, a moderate association cannot be excluded.

In contrast, anti-PLA2R antibody modeled continuously showed an inverse association with remission (OR per 100 RU/mL = 0.836, 95% CI: 0.704–0.992, *p* = 0.041). This indicates that higher baseline antibody levels were associated with lower odds of remission within the observed range, although the borderline *p* value warrants cautious interpretation. Conservative treatment was also associated with lower odds of remission compared with CTX treatment (OR = 0.191, 95% CI: 0.056–0.650, *p* = 0.008). The results of the primary model are presented in Table 2.

**Table 2.** Multivariable logistic regression (model 3).

Variable	OR (95% CI)	*p* value
Anti-PLA2R (per 100 RU/mL)	0.836 (0.704–0.992)	0.041
Albumin (per g/L)	1.035 (0.983–1.090)	0.194
24-h urine protein (per g/d)	0.962 (0.893–1.036)	0.307
Age (per year)	1.003 (0.981–1.024)	0.810
Female (vs male)	1.234 (0.696–2.188)	0.471
Diabetes (yes vs no)	0.675 (0.319–1.427)	0.302
Hypertension (yes vs no)	1.011 (0.590–1.732)	0.969
Cardiovascular disease (yes vs no)	0.530 (0.178–1.578)	0.253
CNI (vs CTX)	0.473 (0.165–1.355)	0.163
RTX (vs CTX)	1.144 (0.358–3.651)	0.820
Other (vs CTX)	0.424 (0.110–1.639)	0.213
Conservative (vs CTX)	**0.191 (0.056–0.650)**	**0.008**
TyG index	0.839 (0.544–1.293)	0.425

OR: odds ratio; CI: confidence interval. Tx_Group categories: CTX as reference. Anti-PLA2R: anti-PLA2R antibody per 100 RU/mL increment.

### 3.3. Anti-PLA2R binary sensitivity analysis

When anti-PLA2R antibody was dichotomized according to the assay threshold, with <2 RU/mL classified as negative and ≥2 RU/mL classified as positive, the association was no longer statistically significant (OR = 0.666, 95% CI: 0.213–2.089, *p* = 0.484). The wide confidence interval is consistent with limited statistical precision, particularly because only 32 patients were classified as anti-PLA2R negative. Importantly, this sensitivity analysis does not invalidate the continuous analysis; rather, it demonstrates that dichotomization at the assay threshold reduces the information available for estimating the association. These results are presented in Table 3.

**Table 3.** Anti-PLA2R binary sensitivity analysis.

Variable	OR (95% CI)	*p* value
Anti-PLA2R positive (vs negative)	0.666 (0.213–2.089)	0.484
Albumin (per g/L)	1.041 (0.987–1.097)	0.137
24-h urine protein (per g/d)	0.964 (0.895–1.038)	0.327
Age (per year)	1.003 (0.981–1.025)	0.794
Female (vs male)	1.160 (0.661–2.037)	0.605
Diabetes (yes vs no)	0.660 (0.314–1.386)	0.272
Hypertension (yes vs no)	1.013 (0.595–1.725)	0.962
Cardiovascular disease (yes vs no)	0.549 (0.184–1.639)	0.282
CNI (vs CTX)	0.483 (0.169–1.379)	0.173
RTX (vs CTX)	1.249 (0.393–3.972)	0.706
Other (vs CTX)	0.386 (0.103–1.453)	0.159
Conservative (vs CTX)	**0.200 (0.059–0.676)**	**0.010**
TyG index	0.773 (0.511–1.171)	0.224

Adjusted for age, sex, diabetes, hypertension, CVD, and treatment group. Anti-PLA2R status: negative (<2 RU/mL) vs positive (≥2 RU/mL). OR: odds ratio; CI: confidence interval.

### 3.4. TyG tertile analysis

TyG was categorized into tertiles according to its observed distribution. The remission rates were 76.0% in T1, 76.0% in T2, and 68.6% in T3. After multivariable adjustment, patients in the highest TyG tertile had lower but statistically non-significant odds of remission compared with those in the lowest tertile (OR = 0.712, 95% CI: 0.369–1.373, *p* = 0.310). The test for linear trend was also non-significant (*p* for trend = 0.310), providing no evidence of a statistically significant dose-response association. These results are presented in Table 4.

**Table 4.** TyG Tertile analysis.

Group	n	Remission rate	OR (95% CI)	*p* value
T1 (Low, reference)	121	76.0%	1 (Reference)	—
T2 (Medium)	121	76.0%	1.098 (0.571–2.111)	0.780
T3 (High)	121	68.6%	0.712 (0.369–1.373)	0.310

Adjusted for anti-PLA2R, albumin, 24-h urine protein, age, sex, comorbidities, and treatment group. *p* for trend = 0.31. OR: odds ratio; CI: confidence interval.

### 3.5. Subgroup and sensitivity analyses

Among patients receiving standard immunotherapy with CTX, CNI, or RTX, TyG remained unassociated with 12-month remission (OR = 0.78, 95% CI: 0.48–1.28, *p* = 0.328). When complete remission was used as the outcome, TyG again showed no statistically significant association (OR = 0.79, 95% CI: 0.52–1.21, *p* = 0.284). Across the primary and sensitivity analyses, the point estimates for TyG were consistently below 1.0, but all corresponding confidence intervals included 1.0. Additional sensitivity analyses: eGFR adjustment (OR = 0.838, 95% CI 0.543–1.294, *p* = 0.425); log-transformed PLA2R (OR = 0.806, 95% CI 0.641–1.012, *p* = 0.063); complete-case analysis (OR = 1.035, 95% CI 0.625–1.728, *p* = 0.894); IPTW (OR = 1.061, 95% CI 0.761–1.479, *p* = 0.725). These findings are summarized in the forest plot (Figure 2) and in Table S2. In sensitivity analyses accounting for treatment switching, the association between TyG and remission remained non-significant when treatment switching was included as a covariate (OR = 0.805, 95% CI 0.518–1.252, *p* = 0.335); the effect of switching itself was not statistically significant (OR = 1.883, 95% CI 0.953–3.719, *p* = 0.068). In the subgroup analysis restricted to patients who did not switch regimens, the OR for TyG was 0.729 (95% CI 0.438–1.213, *p* = 0.223), consistent with the primary analysis. Treatment switch analyses are summarized in Table S3.

**Figure 2.**
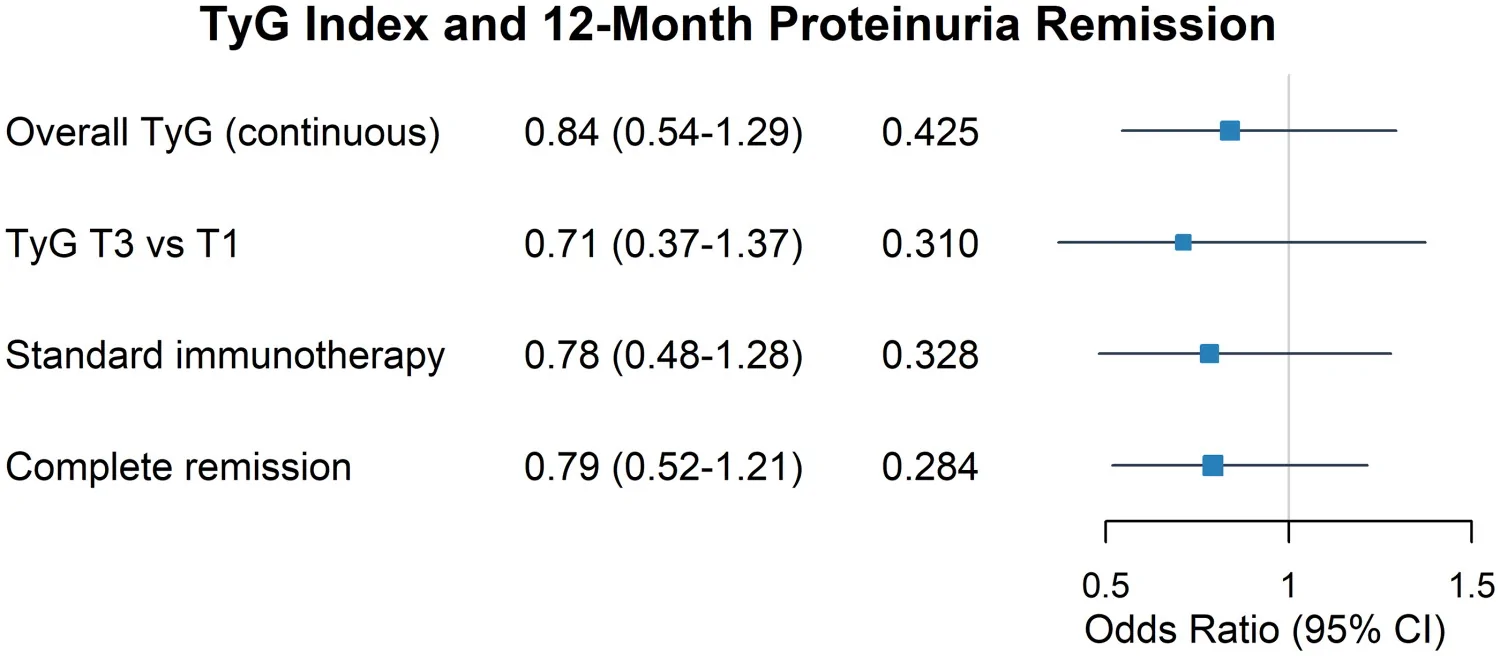
Forest plot of TyG associations across primary and sensitivity analyses. Summary of odds ratios (95% CI) for the TyG index in the primary model, tertile analysis, standard immunotherapy subgroup, and complete remission analysis.

### 3.6. Model performance

The addition of treatment group produced the largest improvement in model discrimination, with mean AUC increasing from 0.637 in Model 1 to 0.714 in Model 2. Adding TyG increased the mean AUC only slightly, from 0.714 to 0.716, whereas mean AIC increased from 407.99 to 409.28. Addition of NLR produced a further small increase in mean AUC to 0.720. These findings suggest that TyG contributed little incremental discrimination beyond the established clinical, immunological, and treatment-related variables. Because AUC and AIC were summarized descriptively across multiply imputed datasets, these comparisons should not be interpreted as formal hypothesis tests. The DeLong test showed no significant difference between Model 2 and Model 3 (Z= −0.636, *p* = 0.525). Model performance metrics are summarized in Table 5. DeLong test and power analysis results are provided in Table S4. ROC curves for all four models are provided in Figure S2.

**Table 5.** Model performance metrics.

Model	Variables	Mean AUC	Mean AIC
Model 1	Base (PLA2R + ALB + UP + age + sex + comorbidities)	0.637	418.22
Model 2	Model 1 + treatment group	0.714	407.99
Model 3	Model 2 + TyG	0.716	409.28
Model 4	Model 3 + NLR	0.720	407.98

AUC and AIC were calculated separately within each imputed dataset and summarized as mean values across the 20 imputations. These are descriptive summaries and should not be interpreted as formal hypothesis tests. AUC: area under the receiver operating characteristic curve; AIC: Akaike information criterion.

## 4. Discussion

### 4.1. Principal findings

In this retrospective cohort study of 363 patients with PMN, baseline TyG was not independently associated with 12-month proteinuria remission after adjustment for established clinical factors, anti-PLA2R antibody, and treatment group. The absence of a statistically significant association was consistent across TyG tertile analysis, restriction to standard immunotherapy recipients, and analysis of complete remission. Furthermore, incorporation of TyG into the conventional model resulted in only a minimal increase in discrimination, with the mean AUC increasing from 0.714 to 0.716. These findings suggest that baseline TyG provides limited additional prognostic information for treatment response in PMN. However, given the confidence interval, a moderate association cannot be excluded.

We also performed additional sensitivity analyses. The eGFR-adjusted model, log-transformed PLA2R, complete-case analysis, and IPTW all yielded consistent results. Post-hoc power analysis showed 91.6% power to detect an OR of 1.5 and a minimum detectable OR of 1.404 (power = 0.80).

Given the previously reported cross-sectional associations between TyG and PMN severity [15], we considered it clinically important to determine whether this readily available marker also predicted treatment response—a question that had not been directly addressed in prior work. Although our findings did not provide statistically significant evidence for an independent association, a moderate association cannot be excluded given the confidence interval.

### 4.2. Comparison with previous studies

Our findings differ from previous studies reporting associations between TyG and renal disease severity. Several studies have linked elevated TyG with chronic kidney disease incidence, progression, or proteinuria [13,14], and a recent study in PMN reported an association between higher TyG and severe proteinuria and reduced renal function [15]. However, these studies primarily evaluated cross-sectional or disease-severity outcomes, whereas our study focused on longitudinal treatment response. Proteinuria remission is influenced not only by baseline metabolic status but also by immunological disease activity and treatment strategy. Therefore, an association between TyG and baseline disease severity does not necessarily imply an independent association with subsequent treatment response.

### 4.3. Potential explanations for the null association

Several mechanisms may explain the absence of a significant association. First, PMN is predominantly an immune-mediated glomerular disease, and disease activity and treatment response are strongly influenced by pathogenic autoantibodies and podocyte injury. Second, TyG may partly reflect metabolic consequences of nephrotic syndrome rather than an independent determinant of treatment response. Nephrotic syndrome can alter lipid metabolism and increase hepatic lipoprotein synthesis, potentially influencing triglyceride concentrations and consequently the TyG index. Third, treatment strategy may exert a substantially stronger influence on remission than baseline metabolic status. In the present analysis, addition of treatment group produced a much larger increase in model discrimination than addition of TyG. Fourth, given that B-cell-mediated mechanisms are the dominant pathogenesis pathway in PMN, and anti-PLA2R antibody is the core pathogenic factor, the prognostic value of TyG may be masked by immunological factors.

### 4.4. Anti-PLA2R antibody and continuous versus binary modeling

An important methodological finding was the difference between continuous and binary modeling of anti-PLA2R antibody. When modeled continuously per 100 RU/mL, higher antibody levels were associated with lower odds of remission. When dichotomized at the assay threshold, however, the association became statistically non-significant. This discrepancy is plausibly related to the limited number of antibody-negative patients and the substantial loss of information caused by dichotomization. Continuous modeling retains variation among antibody-positive patients and therefore generally provides greater statistical efficiency. Nevertheless, the present continuous analysis should not be interpreted as establishing a formal linear dose-response relationship, because no dedicated assessment of the functional form was performed. We have now performed log-transformed and restricted cubic spline analyses, which yielded consistent findings.

For the continuous analysis, values below the assay detection threshold were coded as 1 RU/mL rather than treated as missing. This convention preserved the continuous variable and avoided arbitrary exclusion of patients with undetectable antibody levels. The binary sensitivity analysis, using <2 RU/mL versus ≥2 RU/mL, was conducted specifically to evaluate whether the main conclusion was dependent on this analytical convention.

### 4.5. Clinical implications

Our findings do not support routine use of baseline TyG as an independent prognostic marker for 12-month proteinuria remission in PMN. In contrast, conventional clinical and immunological variables, including treatment strategy, baseline proteinuria, serum albumin, and anti-PLA2R antibody level, remain more directly relevant to assessment of treatment response. Importantly, the present findings should not be interpreted as evidence that metabolic abnormalities are biologically irrelevant to PMN; rather, they suggest that a single baseline TyG measurement may not provide sufficient additional information for predicting short-term remission after accounting for established prognostic factors and treatment.

### 4.6. Strengths

This study has several strengths. First, it evaluated a clinically meaningful longitudinal endpoint rather than only baseline disease severity. Second, the analysis incorporated anti-PLA2R antibody, major clinical variables, and treatment strategy into the multivariable framework. Third, multiple imputation was used to address missing baseline covariates. Fourth, several sensitivity analyses were performed to evaluate the robustness of the TyG finding. Finally, the analysis explicitly distinguished continuous anti-PLA2R modeling from binary sensitivity analysis and prespecified the handling of values below the assay detection threshold. Additional strengths include the use of IPTW to address treatment selection bias and comprehensive sensitivity analyses for anti-PLA2R modeling.

### 4.7. Limitations

Several limitations should be acknowledged. First, the single-center retrospective design limits causal inference and leaves the possibility of residual and unmeasured confounding. Second, treatment allocation was not randomized and may have been influenced by baseline disease severity and clinician preference; we addressed this with IPTW and additionally adjusted for treatment switching, but residual confounding cannot be excluded. Third, TyG and anti-PLA2R antibody were measured only at baseline, and dynamic changes during treatment were not evaluated. Fourth, the binary anti-PLA2R analysis contained relatively few antibody-negative patients, and the EPV was approximately 8; post-hoc power analysis indicated a minimum detectable OR of 1.404, so a moderate association cannot be excluded. Fifth, some patients switched treatment regimens during follow-up, which may introduce misclassification bias; we addressed this by adjusting for treatment switching and performing a subgroup analysis restricted to non-switchers, which yielded consistent results. Sixth, formal risk scores were not available, although sensitivity analyses adjusted for eGFR, proteinuria, and other key prognostic factors.

### 4.8. Future directions

Prospective multicenter studies are needed to validate these findings in larger and more diverse PMN populations. Future research should also evaluate serial TyG measurements, examine whether changes in TyG during treatment are associated with remission, and determine whether TyG has prognostic value for longer-term kidney outcomes. Studies incorporating repeated anti-PLA2R measurements may also clarify whether the dynamic interaction between metabolic status and immunological disease activity has greater prognostic relevance than a single baseline TyG measurement.

## 5. Conclusions

In this retrospective cohort of 363 patients with primary membranous nephropathy, no statistically significant independent association was observed between baseline TyG and 12-month proteinuria remission after adjustment for established clinical, immunological, and treatment-related factors. Adding TyG to the conventional prediction model produced only a minimal improvement in discrimination. However, given the wide confidence interval and a minimum detectable OR of 1.404 in post-hoc power analysis, a moderate association cannot be excluded. Baseline anti-PLA2R antibody level showed an inverse association with remission when modeled continuously, whereas binary classification at the assay threshold provided less precise estimates. Overall, these findings suggest that baseline TyG has limited incremental value for predicting short-term proteinuria remission in PMN.

## Supplementary Material

Supplementary Materials.docx

## Data Availability

The data presented in this study are available on request from the corresponding author. The data are not publicly available due to privacy restrictions.

## References

[1] Couser WG. Primary membranous nephropathy. Clin J Am Soc Nephrol. 2017;12(6):983–997. doi: 10.2215/CJN.11761116. PMC5460716 28550082

[2] Ronco P, Debiec H. Pathophysiological advances in membranous nephropathy: time for a shift in patient’s care. Lancet. 2015;385(9981):1983–1992. doi: 10.1016/S0140-6736(15)60731-0. 26090644

[3] Polanco N, Gutiérrez E, Covarsí A, et al. Spontaneous remission of nephrotic syndrome in idiopathic membranous nephropathy. J Am Soc Nephrol. 2010;21(4):697–704. doi: 10.1681/ASN.2009080861. PMC2844306 20110379

[4] Ruggenenti P, Debiec H, Ruggiero B, et al. Anti-phospholipase A2 receptor antibody titer predicts post-rituximab outcome of membranous nephropathy. J Am Soc Nephrol. 2015;26(10):2545–2558. doi: 10.1681/ASN.2014070640. PMC4587688 25804280

[5] Beck LH, Bonegio RGB, Lambeau G, et al. M-type phospholipase A2 receptor as target antigen in idiopathic membranous nephropathy. N Engl J Med. 2009;361(1):11–21. doi: 10.1056/NEJMoa0810457. PMC2762083 19571279

[6] Kidney Disease: Improving Global Outcomes (KDIGO) Glomerular Diseases Work Group. KDIGO 2021 clinical practice guideline for the management of glomerular diseases. Kidney Int. 2021;100(4S):S1–S276. 10.1016/j.kint.2021.05.021 34556256

[7] Krakowska-Jura K, Kler AN, Wajerowska W, et al. Prognostic factors of proteinuria remission in primary membranous nephropathy. J Clin Med. 2025;14(9):2880. doi: 10.3390/jcm14092880. PMC12072736 40363910

[8] Rojas-Rivera JE, Caravaca-Fontán F, van de Logt A-E, et al. Identification of early predictors of clinical remission in primary membranous nephropathy: a post hoc analysis of the STARMEN trial. Clin Kidney J. 2025;18(9):sfaf256. doi: 10.1093/ckj/sfaf256. PMC12399971 40900938

[9] Bashir K, Asghar MR, Saleem N, et al. Anti-PLA2R antibody reduction as a predictor of treatment response in primary membranous nephropathy: a retrospective study. J Bras Nefrol. 2026;48(2):e20250228. doi: 10.1590/2175-8239-jbn-2025-0228en. PMC12716763 41417012

[10] Valizadeh A, Nikoohemmat M, Ebadinejad A, et al. Metabolic syndrome as a risk factor for the development of kidney dysfunction: a meta-analysis of observational cohort studies. J Diabetes Metab Disord. 2024;23(1):215–227. doi: 10.1007/s40200-023-01348-5. PMC11196551 38932881

[11] Simental-Mendía LE, Rodríguez-Morán M, Guerrero-Romero F. The product of fasting glucose and triglycerides as surrogate for identifying insulin resistance in apparently healthy subjects. Metab Syndr Relat Disord. 2008;6(4):299–304. doi: 10.1089/met.2008.0034. 19067533

[12] Tahapary DL, Pratisthita LB, Fitri NA, et al. Challenges in the diagnosis of insulin resistance: focusing on the role of HOMA-IR and triglyceride/glucose index. Diabetes Metab Syndr. 2022;16(8):102581. doi: 10.1016/j.dsx.2022.102581. 35939943

[13] Tuo J, Li Z, Xie L. Association between triglyceride-glucose index and clinical outcomes among patients with chronic kidney disease: a meta-analysis. BMC Nephrol. 2025;26(1):61. doi: 10.1186/s12882-025-03984-w. PMC11804066 39915738

[14] Sharifi M, Sedgi FM, Soleimani E, et al. Investigating the association between triglyceride-glucose index and risk of chronic kidney disease: a systematic review and meta-analysis. Clin Nutr ESPEN. 2025;69:154–166. doi: 10.1016/j.clnesp.2025.06.028. 40562097

[15] Gao Y-M, Wang Z-H, Deng Z-L, et al. Higher triglyceride-glucose index is associated with severe proteinuria and decreased renal function in patients with primary membranous nephropathy. BMC Nephrol. 2025;26(1):114. doi: 10.1186/s12882-025-04022-5. PMC11884001 40045226

[16] Chen M, Fang Y, Zhang M, et al. Hyperinsulinemia drives glomerular podocyte injury and albuminuria via a self-perpetuating GSK3β-IRS1 insulin desensitization circuit. Metabolism. 2026;179:156571. doi: 10.1016/j.metabol.2026.156571. PMC13715427 41747805

[17] Zaitoon H, Abdul-Ghani M, DeFronzo RA. Insulin resistance and hyperinsulinaemia in kidney disease: mechanisms and metabolic effects. Nat Rev Nephrol. 2026;22(7):474–486. doi: 10.1038/s41581-026-01066-0. 41882295

[18] Inker LA, Eneanya ND, Coresh J, et al. New creatinine- and cystatin C-based equations to estimate GFR without race. N Engl J Med. 2021;385(19):1737–1749. doi: 10.1056/NEJMoa2102953. PMC8822996 34554658

[19] van Buuren S, Groothuis-Oudshoorn K. mice: multivariate Imputation by Chained Equations in R. J Stat Soft. 2011;45(3):1–67. doi: 10.18637/jss.v045.i03.

[20] Rubin DB. Multiple Imputation for Nonresponse in Surveys. New York: John Wiley & Sons; 1987.

