# Supplementary material for: Association between triglyceride-glucose index and treatment response in primary membranous nephropathy: a retrospective cohort study
Source: Ren Fail. 2026 Nov 4;48(1):2751267. doi: 10.1080/0886022X.2026.2751267 (PMC13757830; doi:10.1080/0886022X.2026.2751267)
Supplement: Supplementary file 1 [file IRNF_A_2751267_SM8339.docx]

**Table S1. Missing data proportions**

| Variable | Missing n | Missing (%) |
| --- | --- | --- |
| Age | 0 | 0 |
| Gender | 0 | 0 |
| DM | 0 | 0 |
| Hypertension | 0 | 0 |
| CVD | 0 | 0 |
| Cr (for eGFR) | 0 | 0 |
| ALB | 0 | 0 |
| UP | 0 | 0 |
| PLAR2 | 82 | 22.59 |
| TG (for TyG) | 9 | 2.48 |
| FBG (for TyG) | 27 | 7.44 |
| NEU (for NLR) | 2 | 0.55 |
| LYM (for NLR) | 2 | 0.55 |
| UP_12 (outcome) | 0 | 0 |
| ALB_12 (outcome) | 0 | 0 |

**Table S2. Summary of sensitivity analyses: TyG OR (95% CI) and P values**

| Analysis | TyG OR (95% CI) | P value |
| --- | --- | --- |
| Primary model (Model 3) | 0.839 (0.544–1.293) | 0.425 |
| + baseline eGFR | 0.838 (0.543–1.294) | 0.425 |
| log-transformed PLA2R | 0.806 (0.641–1.012) | 0.063 |
| PLA2R RCS (non-linearity test) | — | 0.729 |
| Complete-case analysis | 1.035 (0.625–1.728) | 0.894 |
| IPTW | 1.061 (0.761–1.479) | 0.725 |
| Treatment switching as covariate | 0.805 (0.518–1.252) | 0.335 |
| No-switch subgroup | 0.729 (0.438–1.213) | 0.223 |

Note: For PLA2R RCS, the P value refers to the non-linearity test, not the TyG OR.

**Table S3. Treatment switching analyses**

**A. Proportion of treatment switching**

| Treatment group | N | Switched, n (%) |
| --- | --- | --- |
| CTX | 29 | 9 (31.0) |
| CNI | 165 | 32 (19.4) |
| RTX | 102 | 25 (24.5) |
| Other | 27 | 4 (14.8) |
| Conservative | 40 | 8 (20.0) |
| Total | 363 | 78 (21.5) |

By remission status: non-remission 17/96 (17.7%), remission 61/267 (22.8%), P = 0.365.

**B. Effect of treatment switching**

| Variable | OR (95% CI) | P value |
| --- | --- | --- |
| Treatment switching (yes vs no) | 1.883 (0.953–3.719) | 0.068 |

**Table S4. Model performance supplement: DeLong test and power analysis**

**A. DeLong test**

| Comparison | Z | P value | Model 2 AUC | Model 3 AUC |
| --- | --- | --- | --- | --- |
| Model 2 vs Model 3 | −0.636 | 0.525 | 0.718 | 0.721 |

**B. Post-hoc power analysis**

| Scenario | Value |
| --- | --- |
| Power for OR = 1.5 | 0.916 |
| Power for OR = 2.0 | 1.000 |
| Minimum detectable OR (power = 0.80) | 1.404 |

**Figure S1. MICE convergence plots**

**Figure S2. ROC curves for all four models (Imputation 1).**
